# A Systematic Review of Yoga as a Supportive Treatment for Children with Attention-Deficit/Hyperactivity Disorder

**DOI:** 10.7759/cureus.63576

**Published:** 2024-07-01

**Authors:** Indushree Manjunath, Varun Channappa, Aditya Karthikeyan

**Affiliations:** 1 Pediatrics, Sapthagiri Institute of Medical Sciences & Research Centre, Bengaluru, IND; 2 General Medicine, Manipal Hospitals, Bengaluru, IND; 3 General Medicine, Ramaiah Medical College, Bengaluru, IND

**Keywords:** nonpharmacological measures, mindfullness, yoga and meditation, pediatrics, yoga therapy, attention-deficit/hyperactivity disorder (adhd)

## Abstract

Attention-deficit/hyperactivity disorder (ADHD) has been one of the leading causes of neuropsychiatric disorders among children, which is why it is extremely essential to have a clear understanding of the disease and the new and upcoming treatment options available. Yoga has been one of the most recently explored and newer techniques that have been employed in the treatment of this disorder. It has become extremely crucial to understand the importance of using supportive treatments in the management of ADHD owing to the innumerable side effects caused by stimulant medication and the growing demands of parental concern about starting their children on stimulant medication at a very young age. Through this review, we would like to shed light on how yoga helps improve the lives of children with ADHD and how it can be used as a supplementary therapy for children with ADHD. After thoroughly screening various articles on this topic, we selected a total of seven articles for this study to highlight the effect that yoga has had on the improvement of ADHD symptoms. Furthermore, we also highlight the impact of such interventions in a family-based environment and how they help improve the immediate environment of a child with this disorder, thereby facilitating the development of a conducive environment for growth. We also highlight how these interventions help control various miscellaneous symptoms among children, such as stress, depression, and anxiety, as these concomitant symptoms are often associated with ADHD. This review article helps explore how yoga has proved to be a very holistic approach to the management of children with ADHD.

## Introduction and background

Mental illness among children is a growing health concern in today’s world, and the burden keeps increasing, debilitating millions of children around the globe every day. The most common neuropsychiatric disorders among children are attention-deficit/hyperactivity disorder (ADHD), autism spectrum disorder, tic disorders, etc. ADHD is one of the leading causes contributing to the disease burden. According to the National Survey of Children’s Health survey conducted in 2020, the prevalence of ADHD among children aged 3-17 years was 9.8% [1]. According to UNICEF, 0.2% of children below the age of 5 and 2.3% of children aged between 15 and 19 who had ADHD also developed developmental disabilities [2].

Children with this disorder often have prolonged periods of inattentiveness, hyperactivity, and/or impulsivity that are usually inappropriate. They frequently cause functional impairment and learning difficulties among children, who almost always require medical treatment [3]. For a child to be diagnosed with ADHD, these symptoms must be present in at least two settings (such as school and home), and the child should have at least six out of 11 symptoms that show inattentiveness and six out of nine symptoms that show hyperactivity and impulsiveness, according to the Diagnostic and Statistical Manual of Mental Disorders (DSM)-5 criteria [4].

This disorder has a chronic course and often causes problems even as a child grows into adulthood. The most common comorbidities of ADHD include the risk of substance misuse, dysregulated eating, and neurological comorbidities such as migraine; all these issues significantly hinder the normal development of the child [5].

Taking a holistic, multimodal approach while treating a child with ADHD is always considered the most beneficial. This includes targeting all aspects of care such as parent counseling, behavioral therapy for the child, environment-centered interventions, and pharmacological intervention, providing a well-rounded approach while treating a child with ADHD [5].

The nonpharmacological modalities currently used to treat ADHD are cognitive behavioral therapy and neuropsychological treatment (such as goal-oriented therapy and exposure therapy). The use of mindfulness techniques as a complementary approach is receiving a lot of attention and has turned out to be extremely promising. Mindfulness meditation practices and yoga aim to cultivate awareness among children and develop focus through various breathing exercises and postures (asanas). Recent neuropsychological research shows that mindfulness training can be used as a potential treatment for children with ADHD, as it helps them to be attentive and reduce impulsivity [6]. Various techniques have been experimented with, such as mindful eating, mindful walking, body scans, and mindful movements. These practices help an individual focus on subtle aspects of common everyday activities and help develop a sense of focus and control over one’s mind, body, and emotions. Most mindfulness practices use the breath as a common focus point that individuals are instructed to focus on whenever they experience distractions. Studies suggest that mindfulness techniques result in changes in the gray matter concentration of the prefrontal cortex that regulates both attention and emotions, which is often abnormal in patients with ADHD [7].

Yoga is considered to be a mindfulness technique that involves both the mind and the body. People are usually taught to focus through the combination of various postures called asanas and breathing techniques. The holistic goal of yoga is to foster both physical and mental health while also focusing on the subtle spiritual aspects of life. The use of yoga as an alternative and as supportive therapy for children is extremely appealing owing to the complicated nature of pharmacotherapy used in the treatment of ADHD and the side effects of the stimulant medication, which is to date the most effective medication. Our goal is to highlight the importance of this global phenomenon in improving the quality of life and reducing symptoms in children with ADHD and to help highlight the benefits of including yoga as an intervention alongside stimulant and nonstimulant medication in the treatment of ADHD.

## Review

Methods

**Figure 1:**
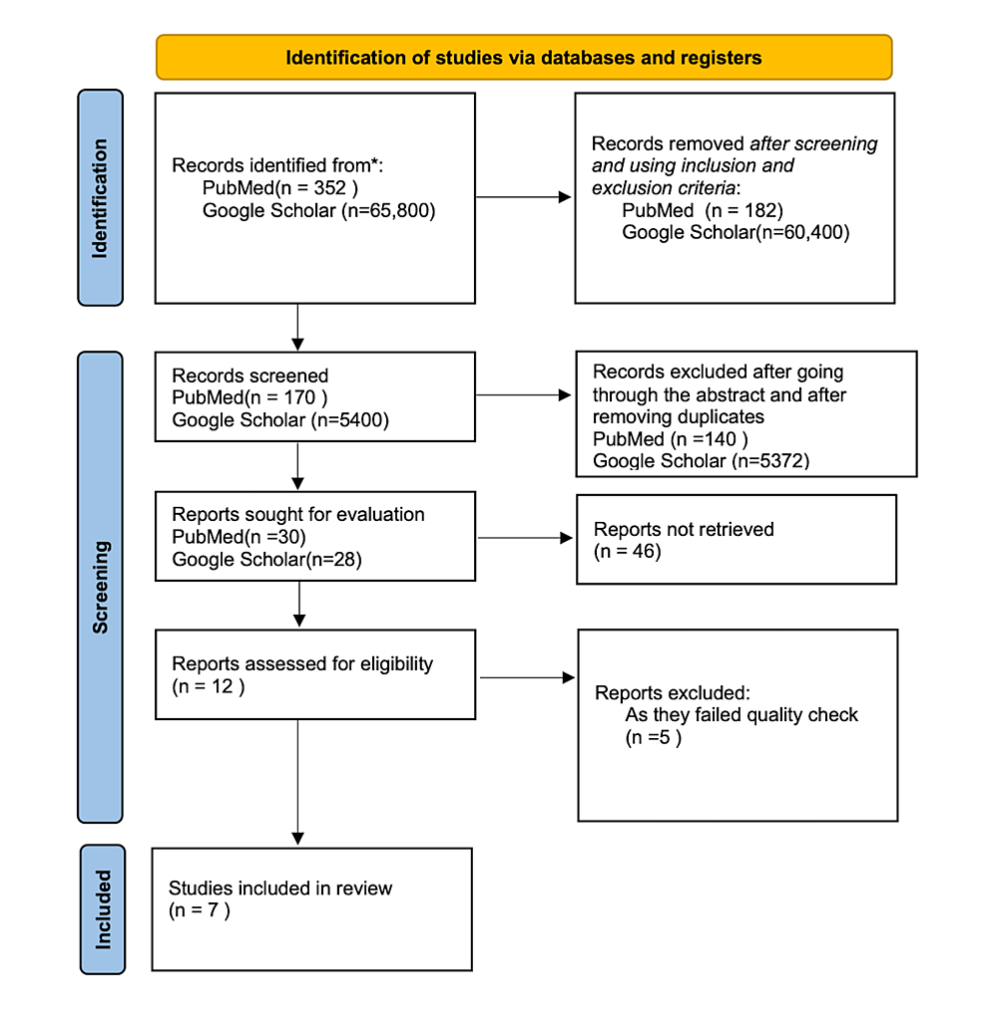
PRISMA 2020 flow diagram showing the article selection process for this review PRISMA, Preferred Reporting Items for Systematic reviews and Meta-Analyses

We have reported the following information in our review article based on the Preferred Reporting Items for Systematic reviews and Meta-Analyses (PRISMA) 2020 guideline for reporting systematic reviews, as shown in Figure 1 [8].

Search Strategy

We conducted extensive electronic searches on PubMed and Google Scholar from April 2024 to May 2024. The keywords we narrowed down to include “ADHD”, “Children”, “yoga”, “therapy”, “mindfulness”, ’treatment” and therapeutics”, and we used them to look up articles in different databases. We conducted most of our search on PubMed using PubMed Advanced using the keywords mentioned above in the following combination: “ADHD AND mindfulness”, “ADHD AND yoga”, “ADHD AND yoga AND therapy”, ((yoga) AND (attention)) AND (children) and (ADHD) AND (nonpharmacological).

Each article’s title and abstract were screened, keeping our predetermined inclusion and exclusion criteria in mind. We selected a total of seven articles for full-text reading and screening. Where full texts were not available, the articles were screened using the abstracts, and attempts were made to obtain the complete article for screening. All authors reviewed the studies individually, and a final collective decision was taken regarding which articles should be included in this review.

Inclusion Criteria

The articles we included were clinical trials and randomized control trials, published in English from 2000 until May 24, 2024. Children between the age groups of two and 18 were included in this review.

Exclusion Criteria

The articles we excluded were published in English before 2000 and after May 24, 2024. Children not between the age groups of two and 18 were excluded from this review. Any article that was not a clinical or randomized control trial was also excluded.

Results

Using the keyword mentioned above, we searched PubMed and Google Scholar. We found 352 articles on PubMed and 65,800 on Google Scholar. Among these articles, 170 articles on PubMed and 5,400 articles on Google Scholar fit our inclusion/exclusion criteria. They were further screened, and duplicates were removed. We only selected randomized control trials and clinical trials for our review. We did a quality assessment of the 12 articles that were eligible and selected seven articles for review. Table 1 shows a summary of all the articles included in the review.

**Table 1:** Summary of all the articles included in the review ADHD, attention-deficit/hyperactivity disorder; ANT, Attention Network Test; SDQ, Strengths and Difficulties Questionnaire; SWAN, Strengths and Weaknesses of ADHD Symptoms and Normal Behavior

Study	Sample size	Age range	Intervention	Duration	Outcome measures	Key findings
Telles et al. (2019) [9]	61	11-12 years	Yoga and breathing practices	3 days	Attention and anxiety	Significant improvement in attention and anxiety
Saxena et al. (2020) [10]	174	14-15 years	Hatha yoga	12 weeks	SWAN and Perceived Stress Scale	Reduction in inattention and hyperactivity
Farahani et al. (2017) [11]	80	9-13 years	Superbrain yoga	1 month	Conners’ Parents Rating Scale	Decrease in ADHD symptoms
Cohen et al. (2018) [12]	23	3-5 years	Yoga practice	6 weeks	ADHD Rating Scale-IV and SDQ	Improvement in attention and impulsivity
Jensen and Kenny (2004) [13]	14	8-13 years	Yoga program	20 sessions	Conners’ Parent and Teacher Rating Scales	Reduction in symptoms, mood swings, and temper tantrums
Santonastaso et al. (2020) [14]	32	7-11 years	Mindfulness meditation	8 weeks	Neuropsychological tests and academic measures	Improvement in neuropsychological functions and behavior
Lo et al. (2017) [15]	100 families	5-7 years	Family-based mindfulness	6 weeks	SWAN, Child Behavior Checklist, and ANT	Improvement in attention and behavior and reduction in parental stress

Quality Check

All the selected articles finally went through a quality check. All the randomized control trials were assessed by the Cochrane risk of bias tool, and the Newcastle-Ottawa Tool Scale was employed to evaluate clinical trials.

Discussion

Yoga as Therapy for Attention Disorders in Children

We have included three randomized controlled trials in our review to talk about yoga’s role in improving the lives of children with attention disorders.

The study conducted by Telles et al. evaluated 61 pre-teen children aged 11 and 12 years, among which 25 were girls [9]. They studied the immediate effect of yoga and breathing practices on attention and anxiety in pre-teen children. They suggested that just 18 minutes of focusing on one’s breath, high-frequency breathing techniques, and sitting in a quiet setting improved a child’s focus. Their scores were significantly better when they performed an attention-based task following the yogic practices. Attention among participants was assessed using the six-letter cancellation task, and Spielberger’s State Trait Anxiety Inventory was used to measure anxiety before and after the yoga practice [9]. This study randomly assigned the children participating into three groups: Group A, Group B, and Group C. Group A performed high-frequency yogic breathing, breath awareness, and quiet sitting on three consecutive days. Group B performed breath awareness, high-frequency yogic breathing, and quiet sitting on three successive days. Group C performed quiet sitting, breath awareness, and high-frequency yogic breathing on three successive days [9]. All groups took the assessments mentioned above before and after the practices. The data collected was analyzed, and the results showed that attention among children improved after high-intensity yoga breathing alone. This study also showed that the decrease in anxiety and attention improvements was more prominent among girls than boys. This study concluded that yoga-based breath awareness techniques helped improve attention and alleviate anxiety among school-aged children [9]. This study is particularly interesting, as ADHD is also a disorder among kids that focuses on a lack of attention and focus. Hence, the findings can be correlated to this subgroup of patients.

Another study conducted by Saxena et al. evaluated the benefits of yoga and meditation on attention, hyperactivity, and stress in high school children. The objective was to study the impact of Hata yoga on improving attention and alleviating stress in high school children [10]. A total of 174 ninth graders between the ages of 14 and 15 were enrolled in the study, among which 123 students were assigned to the yoga group and 51 students to the control group [10]. The Hata yoga intervention performed by the students was a 25-minute yoga and meditation session (18 minutes of asanas and seven minutes of meditation) performed twice a week. This was continued for 12 weeks. At the end of the 12 weeks, the yoga group was asked to fill out a questionnaire called the Strengths and Weaknesses of ADHD Symptoms and Normal Behavior (SWAN), an 18-item questionnaire to assess focus levels among the children. The Perceived Stress Scale, a 10-item questionnaire, was used to assess the stress levels among the children as well [10]. The data was analyzed at the end of the study, showing a significant reduction in inattention and hyperactivity among children who practiced Hata yoga. Hence, the introduction of a small 20- to 25-minute Hata yoga practice in an ADHD child’s routine can significantly decrease symptoms of inattention [10].

The randomized control trial conducted by Farahani et al. on the effectiveness of superbrain yoga on children with hyperactivity disorders had some interesting findings as well [11]. The study included 80 school-aged children between the age groups of nine and 13 who had a prior diagnosis of ADHD. A child psychiatrist filled out the Conners’ Parents Rating Scale, which had 48 questions at the beginning and end of the study, and the scores were compared [11]. The superbrain yoga practice was taught to both children and parents, and after the training, parents were instructed to make their children perform the same at home under supervision. The children performed this practice every day at home for two minutes, and this was followed up by a research partner every week by making phone calls to the parents [11]. At the end of the month, the children were evaluated using the same questionnaire as mentioned above. At the end of the study, results showed that the severity of ADHD symptoms had reduced significantly before and after the intervention [11]. The improvement was observed in a range of symptoms, such as psychosomatic problems, conduct problems, and impulse control. Hence, superbrain yoga was suggested as a complementary treatment along with pharmacotherapy for the management of ADHD in primary school children [11].

ADHD and Yoga

Cohen et al. conducted a randomized control trial on the effects of yoga on attention, impulsivity, and hyperactivity in preschool children with ADHD [12]. The study evaluated 23 children aged three to five years. They employed the ADHD Rating Scale-IV Preschool Version and Strengths and Difficulties Questionnaire to assess the participants. They further divided the participants into two groups based on random allocation [12]. The groups were labeled as the control group and the group performing the intervention. The group performing the yoga practice did so for six weeks. After six weeks elapsed, the two groups switched, and the intervention was now performed on the group that previously served as control. A yoga practice was designed for the children to perform in the yoga group or intervention group [12]. The groups were assessed using the questionnaire at four points: at the beginning of the study, in between before the switch, when the second group performed the intervention as well, and three months after the end of the intervention. The results were analyzed, and it was found that group one and group two did not show any significant differences after the first assessment before the study began [12]. The KiTAP Tasks of Attention were used and showed that children had faster reaction time, were able to focus well, were less distracted, and had fewer errors of omission after the intervention. The study concluded that yoga could be used as an effective tool for children with symptoms of ADHD, and children who had more profound symptoms had a greater level of improvement. However, studies with a larger number of participants will help better evaluate the role that yoga can play as an intervention for children with ADHD [12]. 

Jensen and Kenny conducted a study to analyze the effects of yoga on the attention and behavior of male children with ADHD [13]. They selected a group of 14 boys between the ages of eight and 13, and they were enrolled. The boys were randomized into two groups: one group was the yoga group, and the other served as control. After completing a 20-session-long program, the participants switched groups. Out of the 14 boys, six who were originally in the yoga intervention stayed in their group, five boys who initially served as control joined the yoga group, and the remaining three boys continued with the yoga intervention [13]. Only boys who were diagnosed according to DSM-IV criteria by experienced pediatricians were included in the study. At the end of the study, a total of 11 boys completed the yoga intervention, and eight boys served as controls. Techniques such as postural training, respiratory training, relaxation training, and concentration training were included in the program. The Conners’ Parent and Teacher Rating Scales were used to assess behavioral changes [13]. It revealed that there was a significant difference between the symptoms among children with ADHD before and after the intervention. The children experienced fewer mood swings, crying episodes, and temper tantrums. Hence, the study revealed that yoga had a positive, calming effect on the participants while significantly reducing their symptoms. Unfortunately, the Test of Variables of Attention, a continuous performance test that was employed to assess attention and impulsivity among children participating in the study, did not provide any valuable results, and the actigraph that assessed levels of hyperactivity had many technical difficulties as well. However, at the end of the study, it was concluded that the use of yoga as a complementary tool in the management of children with ADHD was extremely effective, especially toward the end of the day when the child is at home and in a more controlled environment. The study also confirmed that yoga was most useful to control symptoms when the child was off medication or the effect of the medication had worn off toward the end of the day [13].

Table 2 shows the effect of yoga and yoga-based interventions on various symptoms of ADHD.

**Table 2:** Effects of yoga on various symptoms of ADHD ADHD, attention-deficit/hyperactivity disorder

ADHD symptom	Intervention	Study	Sample size	Improvement level
Inattention	Yoga	Saxena et al. (2020) [10]	174	High
Hyperactivity	Yoga	Saxena et al. (2020) [10]	174	Moderate
Impulsivity	Yoga	Cohen et al. (2018) [12]	23	Significant
Anxiety	Yoga and breathing	Telles et al. (2019) [9]	61	Moderate
Behavioral issues	Yoga	Jensen and Kenny (2004) [13]	14	Significant
Stress	Hata Yoga	Saxena et al. (2020) [10]	174	High

ADHD and Mindfulness

A clinical trial was conducted by Santonastaso et al. on applying mindfulness-oriented meditation. Thirty-two children, aged between seven and 11 years and diagnosed with ADHD by an experienced child psychiatrist using DSM-5 criteria, were participants in this study [14]. The information collected before and after the intervention includes neuropsychological and academic measures and behavioral, emotional, and mindfulness ratings. Thirty-five children with ADHD were initially included in the study on a waiting list. Experienced developmental psychiatrists and neurophysiologists examined them all, and a diagnosis of ADHD was made based on the DSM-5 criteria. The baseline assessment was conducted by two psychologists who were blinded to the intervention. Immediately after the intervention, since two children disagreed with participating, we were left with a group of 32. Subsequently, they were divided into two groups of 16 children each [14]. One group underwent mindfulness-oriented meditation training, while the other was involved in the emotional education program. Evaluations were made after the training by psychologists who were blinded to the intervention. The mindfulness-oriented meditation was conducted twice a week for eight weeks. The duration of the sessions gradually increases to a maximum of 30 minutes per session at the end of the study period [14]. This program involved techniques such as mindfulness breathing, being mindful of body parts, and being mindful of one’s thoughts and emotions. Each activity was structured in the form of games to facilitate participation and enhance involvement among the children. They were also instructed to maintain meditation diaries. On the other hand, the emotional education program had a similar structure and spanned eight weeks [14]. This group had to read the book “Six Pixies in My Heart” and comment on it. At the end of this activity, the children learned the importance of positive and negative emotions and how to pay attention to one’s emotions and effectively interpret them. At the end of the study, the two groups were compared, and it was found that mindfulness meditation practices, even when performed for just eight months, had a profound impact on children with ADHD; they helped promote neuropsychological changes and control behavior symptoms among them [14].

A randomized control trial was conducted by Lo et al. on the effects of family-based mindfulness interventions on managing ADHD [15]. A total of 123 families with children aged between five and seven years who either met or exceeded the cutoff of the SWAN Rating Scale were included in the study. Of the initial 123, 23 families were excluded as they did not meet the cutoff. Among the remaining children, 74 already had a diagnosis of ADHD. The 100 families were randomized into two groups: the intervention group and the waitlist control group. The parent program lasted for six weeks, with each session lasting 1.5 hours based on a protocol prepared by one of the authors [15]. The child program lasted one hour and followed the child mindfulness program, “Mindfulness Matters.” A joint 30-minute activity was incorporated at the end of the parent program. The following three variables were used to assess the outcome of this intervention among children: SWAN, Child Behavior Checklist, and Child Attention Network Test (ANT). The adults participating in the study were tested using the following measures: Parenting Stress Index (PSI), Adult ADHD Self-Report Scale, Interpersonal Mindfulness in Parenting, Interpersonal Mindfulness in Parenting, and Parent Heart Rate Variability. One measure called the MBI-TAC was employed to assess treatment fidelity [15]. The study revealed that there was a profound improvement in the child’s attention and behavior after the mindfulness intervention. The benefits for the parents were also evident based on the PSI subscales. It showed that parental stress reduction contributed to maintaining a nurturing environment for children with ADHD [15]. The study also showed a significant improvement in the ANT, which suggested that there was a significant improvement in the attention span among the participants. However, they did not find any difference in heart rate variability among the adult participants in the study. Hence, it was concluded that mindfulness-based interventions were a very important tool in creating a positive family environment to nurture children with ADHD [15].

Table 3 summarizes the effect of mindfulness-based therapy on children with ADHD.

**Table 3:** Effects of mindfulness-based techniques on ADHD ADHD, attention-deficit/hyperactivity disorder

Technique	Description	Study	Sample size	Key findings
Mindful breathing	Focus on breathing to reduce distractions	Santonastaso et al. (2020) [14]	32	Improved attention and emotional regulation
Mindful body scan	Awareness of different body parts	Santonastaso et al. (2020) [14]	32	Enhanced control over behavior
Superbrain yoga	Specific yoga postures are believed to enhance brain function	Farahani et al. (2017) [11]	80	Reduced hyperactivity and conduct problems

Limitations

Our review’s limitations include using only clinical trials and randomized control trials. All articles published before 2000 were omitted; hence, the data in this article only holds good for making inferences after this period. We would also like to mention that, considering the limited number of research articles on the use of yoga in the management of ADHD, more research is required in this field to draw firm conclusions about the benefits and drawbacks of the use of yoga as an alternative form or as supportive therapy for children with ADHD.

## Conclusions

The primary goal of our study was to evaluate the benefits of using yoga as an alternative form of therapy and treatment for children diagnosed with ADHD. Using the studies mentioned above, we were able to show that there is a strong association between the use of yoga and improvements in ADHD symptoms among children of various age groups. It is not only the use of yoga that has proven to be effective, but also the use of yoga-based mindfulness techniques that have been shown to benefit these children. Moreover, our article also indicates that combining various yoga-based therapies with conventional medications helps drastically improve the symptoms of ADHD. Furthermore, the use of yoga as a family-based intervention created a supportive environment that fostered the development and well-being of these children, helping them thrive. Although ADHD is a chronic health condition that debilitates many children, the advent of supportive therapies with minimal adverse effects that complement conventional treatments holds a very promising future for the treatment of children with ADHD.
